# The Anti-Inflammatory Effect of Guchangzhixie-Pill by Reducing Colonic EC Cell Hyperplasia and Serotonin Availability in an Ulcerative Colitis Rat Model

**DOI:** 10.1155/2017/8547257

**Published:** 2017-08-06

**Authors:** Yong Yang, Xianwei Zhu, Yifei Qin, Guihai Chen, Jing Zhou, Lu Li, Jianjun Guan, Li Ma, Yanyan Xue, Chenhui Li

**Affiliations:** ^1^College of Basic Medicine, Shaanxi University of Chinese Medicine, Xi'an-Xianyang New Economic Zone, Xianyang, Shaanxi 712046, China; ^2^Innovation Research Centre of Acupuncture Combined with Medicine, Shaanxi University of Chinese Medicine, Xi'an-Xianyang New Economic Zone, Shaanxi 712046, China

## Abstract

Ulcerative colitis (UC) is one of the major types of inflammatory bowel diseases (IBD). Abnormal colonic enterochromaffin (EC) cell hyperplasia and serotonin availability have been described in UC. Guchangzhixie-pill (GCZX-pill), a Chinese herbal formula composed of six herbs, is modified based on a traditional formula (Jiechangyan-pill) for inflammatory and ulcerative gastrointestinal disorders. This study aims to investigate the anti-inflammatory effect and the underlying mechanisms of GCZX-pill on trinitrobenzene sulfonic acid- (TNBS-) induced UC in rats. After orally administrating a GCZX-pill to UC rats for 14 days, the results of the inflammation evaluation, such as disease activity index (DAI), macroscopic score (MS), myeloperoxidase (MPO) activity, and other methods, suggested that the GCZX-pill showed remarkable anti-inflammatory results in UC rats. In addition, the abnormal EC cell numbers, colonic tryptophan hydroxylase (TPH) expression, and serotonin (5-HT) contents in TNBS-induced UC rats were significantly reduced by the GCZX-pill. This data demonstrates that the GCZX-pill can attenuate the inflammation in UC rats and the anti-inflammatory effect of the GCZX-pill may be medicated by reducing colonic EC cell hyperplasia and 5-HT availability.

## 1. Introduction

Ulcerative colitis (UC) is one of the major types of inflammatory bowel diseases (IBD) that arises as a result of the interaction between environmental and genetic factors leading to immunological responses and inflammation in the intestine. It is characterized by inflammation of the mucosa of the intestinal tract, causing ulceration, edema, bleeding, and fluid and electrolyte loss [1, 2]. In recent years, progress has been made in understanding the pathogenesis of UC and in developing improved therapeutic approaches which include treatment with immune-modifying agents (mercaptopurine or azathioprine) or one of the anti-TNF agents earlier in the course of UC. Nevertheless, many side effects, which were caused by these therapeutic approaches, have been reported [3–6]. Conversely, in clinical practice, many Chinese herbal formulas as an alternative treatment modality for the treatment of UC acquired an observably therapeutic effect [7, 8].

Colonic changes in UC are characterized by ulcerative lesions accompanied by a prominent infiltrate of immune cells as well as alteration in serotonin (5-hydroxytryptamine; 5-HT) [9, 10]. 5-HT, as neurotransmitter and paracrine signalling molecule in gastrointestinal tract (GIT), not only plays important roles in initiating peristaltic, secretory, vasodilatory, vagal, and nociceptive reflexes, but can also activate the immune cells and regulate the GIT inflammation and intestinal pathophysiology as has been reported in past studies [11, 12]. 5-HT is synthesized and released from enterochromaffin (EC) cells and the tryptophan hydroxylase (TPH) which in EC cells is the rate-limiting enzyme in the 5-HT synthesis process [13]. Moreover, colonic EC cell hyperplasia has been reported recently. The underlying mechanisms of EC cell hyperplasia in UC are unknown, but they are considered to have close correlation with CD4^+^ T lymphocytes, especially the T_h1_/T_h2_ balance [14, 15]. These findings indicated that the targeting effect of EC cells and 5-HT is a therapeutic strategy for UC.

Guchangzhixie-pill (GCZX-pill) is modified based on a traditional formula (Jiechangyan-pill) for diarrhea, abdominal pain, UC, and irritable bowel syndrome (IBS). It contains* Prunus mume*,* Zingiber oj-jicinale* Rosc.,* Coptis chinensis* Franch.,* Pericarpium Papaveris*,* Radix Aucklandiae*, and* Corydalis yanhusuo* [16–18]. Past studies indicated that GCZX-pill relieved symptoms in UC or IBS patients. In recent years, more and more clinicians have successfully applied GCZX-pill in inflammatory and ulcerative gastrointestinal disorder prevention and treatment and have received a satisfactory clinical outcome [19–22]. Nevertheless, the effect of GCZX-pill in UC requires further clinical evidence and definitive mechanisms of action.

In this study, an experimental UC rat model induced by trinitrobenzene sulfonic acid (TNBS) was used. After treatment of the TNBS-induced UC rat model with GCZX-pill, the changes of colonic EC cells number, TPH expression, 5-HT, and some cytokine productions were investigated to gain an understanding of the pharmacological mechanism of the GCZX-pill.

## 2. Materials and Methods

### 2.1. Materials

All the herbs in GCZX-pill were provided by the Affiliated Hospital of Shaanxi University of Chinese Medicine (Xianyang, Shaanxi, China) and extracted according to the standard methods recommended by the Chinese Pharmacopoeia (2010). In this study,* Prunus mume* was collected from Dujiangyan, Sichuan, China;* Zingiber oj-jicinale* Rosc. was collected from Hancheng, Shaanxi, China;* Coptis chinensis* Franch. was collected from Shizhu, Chongqing, China;* Pericarpium Papaveris* was collected from Nongken, Gansu, China;* Radix Aucklandiae* was collected from Dali, Yunnan, China; and* Corydalis yanhusuo* was collected from Chenggu, Shaanxi, China. Currently, these dried powdered herbs are being stored in the Innovation Research Centre of Acupuncture Combined with Medicine of Shaanxi University of Chinese Medicine. A total of 100 g of the herbs was mixed and macerated in water for 30 min, subsequently decocted for 60 min three times, and rinsed 10 times (v/w) with distilled water. Then, the final residues were filtered using microfilter, concentrated, and then made into dried powder. Approximately 12.4 g of powder was obtained. The concentration of berberine hydrochloride in the product was measured (7.901 mg/g) for quality standard research according to the Drug Standard of Ministry of Public Health of the People Republic of China (WS3-B-3870-98) [16–18].

### 2.2. Animals

Male Sprague-Dawley rats (weighing approximately 220 g) were housed in polycarbonate cages in isolators under a 12 h light/12 h dark cycle, with controlled temperature 21 ± 3°C and humidity 60%. In addition, rats were fed ad libitum with standard food and water. All animal care and experimental procedures complied with the regulations of Shaanxi Administration Rule of Laboratory Animal (authorization number: #109228/2014). All experiments in this study were approved by the Laboratory Animal Ethics Committee at the Shaanxi University of Chinese Medicine.

### 2.3. Induction of Experimental UC Rat Model

UC rats were induced by the intracolonic administration with TNBS [23, 24]. After being fasted for 24 hours, the rats were deeply anesthetized with chloral hydrate (350 mg/kg, i.p.). Rats were given 0.9% saline solution or TNBS-solution (30 mg/kg, soluble in 50% ethanol) via PE90 tubing inserted to 8 cm from the anus. Then, the rats given 0.9% saline solution were divided into the control group and treated with 0.9% saline solution. The UC rats were randomly divided into 5 groups (*n* = 12) and treated with 0.9% saline solution (i.p., TNBS group), or para-chlorophenylalanine (pCPA, TPH inhibitor, 150 mg/kg/d in 0.9% saline solution, i.p., pCPA group), or gavage administration of GCZX-pill (group 4–6) at a dose of 2.4, 4.8, and 9.6 g/kg/d (dissolved in water) once a day for 14 days.

### 2.4. Macroscopic Evaluation of Inflammation

The macroscopic colonic inflammations in the TNBS-induced UC rats before and after treatment with pCPA or GCZX-pill were evaluated by the methods of disease activity Index (DAI), macroscopic score (MS), colon thickness, and colonic edema [25–29]. The protocols are briefly described below.

#### 2.4.1. DAI

The DAIs of the rats were based on body weight loss, stool consistency, and rectal bleeding and scored blindly by three investigators. The scores were quantified as follows: stool consistency: 0 (normal), 1 (soft), and 2 (liquid); body weight loss: 0 (<5%), 1 (5–10%), 2 (10–15%), 3 (15–20%), 4 (20–25%), and 5 (>25%); and rectal bleeding: 0 (absent) and 1 (present).

#### 2.4.2. MS

After treatment of pCPA or GCZX-pill, the rats were passively euthanized with carbon dioxide. The colon was visualized and rapidly excised in its entirety and placed in a Petri dish containing sterile saline solution. The colons were opened along the mesenteric line and immediately evaluated for the presence of colonic inflammation. MS was quantified by a blinded investigator. The criteria were quantified as follows: the presence of points of stenosis and hypertrophic zones (0, absent; 1, 1 stenosis; 2, 2 stenoses; 3, more than 2 stenoses), mucus (0, absent; 1, present), adhesions (0, absent; 1, 1 adhesion between colon and other intra-abdominal organs; 2, 2 adhesions; 3, more than 2 adhesions), intraluminal hemorrhage (0, absent; 1, present), erythema (0, absent; 1, area < 1 cm^2^; 2, area > 1 cm^2^), and ulceration and necrosis zones (0, absent; 1, area < 1 cm^2^; 2, area > 1 cm^2^).

#### 2.4.3. Colon Thickness

After the quantitation of MS, the length and weight of colon were measured, while weight/length ratio was calculated to estimate colon thickness.

#### 2.4.4. Colonic Edema

After the estimation of colon thickness, 1 cm long colon segments were cut and weighed immediately as the wet weight (WW) of colon. Then, the colon segments were placed in an oven set to 60°C for 3 days and weighed as the dry weight (DW) of colon. The (WW − DW)/DW ratio was calculated to estimate colonic edema.

### 2.5. Histological Evaluation

Three colon segments from each rat were used for hematoxylin-eosin staining by random choice. Then, the colons were fixed in 4% buffered formaldehyde and embedded in paraffin. Sections were cut to a thickness of 5 *μ*m and stained with hematoxylin-eosin. The sections were examined under a light microscope (Olympus CKX41 31PHP) by a person unaware of the treatment.

### 2.6. Colonic MPO Activity Assay

The colonic samples were homogenized and centrifuged (40,000*g* for 15 min at 4°C) for obtaining the supernatants. The supernatants were diluted (1 : 10) with distilled water. The diluted supernatants (100 *μ*l) were mixed with a buffer solution containing o-dianisidine (0.167 mg/ml, 900 *μ*l) and H_2_O_2_. Each assay was performed in duplicate and the rate of change in absorbance was measured spectrophotometrically at 470 nm. One unit is defined as the amount of MPO enzyme that can metabolize 1 *μ*mol of H_2_O_2_ per min at 25°C. Data were normalized with edema values and expressed as U/g of dry weight tissue [30, 31].

### 2.7. Enzyme-Linked Immunosorbent Assay (ELISA)

The content of 5-HT (MBS725497, MyBioSource, CA) and the cytokine levels of TNF-*α* (MBS2507393, MyBioSource, CA), IFN-*γ* (ab46107, Abcam, UK), and IL-10 (ab100764, Abcam, UK) in the colon tissue were assayed by ELISA. The samples were measured according to the manufacturer's protocol. Absorbance at 450 nm in each well was measured using a spectrophotometer [32].

### 2.8. EC Cell Counting

An evolutionary Fontana-Masson staining was used for EC cell counting [33]. Briefly, the colonic sections were deparaffinized and rehydrated for Fontana-Masson staining (ab150669, Abcam, UK). The sections were incubated in ammoniacal silver solution (1 h, 60°C), gold chloride solution (0.2%, 30 seconds), sodium thiosulfate solution (5%, 2 min, at room temperature), and nuclear fast red solution successively. At last, the sections were dehydrated with alcohol and mounted in synthetic resin. Five random fields at 200x magnifications were counted in each section by a researcher blinded to the treatment; the number of EC cells per mm^2^ of mucosa was quantified using Image J NIH software.

### 2.9. Western Blot Analysis

The expression of colonic TPH was analyzed by western blot analysis. Briefly, the total protein of the colon was extracted and quantified. Then the samples containing 30 *μ*g of protein were boiled for 5 min and subjected to SDS-PAGE electrophoresis and then transferred to PVDF membranes. The PVDF membranes were incubated in a blocking buffer and then incubated with anti-tryptophan hydroxylase antibody (ab46757, Abcam, UK) for 1 night at 4°C. Subsequently, incubation with secondary antibodies labeled alkaline phosphatase (W3960, Promega, USA). The immunoblots were detected by western blue and quantified using the Image J program [34].

### 2.10. Immunohistochemistry Staining for SERT

The colonic sections were deparaffinized and rehydrated for immunohistochemistry staining for SERT. After the sections were quenched in methanol: 30% H_2_O_2_ (9 : 1) for 10 min and microwaved for 10 min in citrate buffer (10 mM, pH 6.0), the colonic sections were blocked in 3% BSA and incubated with anti-Serotonin Transporter (SERT) antibody (S1001-25B, USBiological, US) at 1 : 800 dilution overnight at 4°C, respectively. Followed by incubation with 2nd antibody conjugated with biotin (SA1022, Boster, PRC) and streptavidin conjugated with POD for 2 hours respectively. The immunoblots were detected by diaminobenzidine (DAB) (AR1022, Boster, PRC). The sections were examined under light microscope by a person unaware of the treatment and analyzed using Image J NIH software.

### 2.11. Statistics

All data are presented as mean ± standard error of the mean (SEM). Statistical analysis was conducted using SPSS 15.0 Software (SPSS Inc., Chicago, IL, USA). The differences between groups were considered significant using a one-way analysis of variance (ANOVA). After testing for homogeneity of variance, data of EC cell counting, TPH, cytokines levels, 5-HT, and 5-HIAA in colon were compared using Bonferroni's posttest. Nonparametric Kruskal–Wallis analysis followed by Dunn's posttest was applied for statistical comparison of DAI and MS. Differences were considered significant when *P* < 0.05.

## 3. Results

### 3.1. Macroscopic Evaluation of Inflammation before and after Being Treated with GCZX-Pill

**Figure 1:**
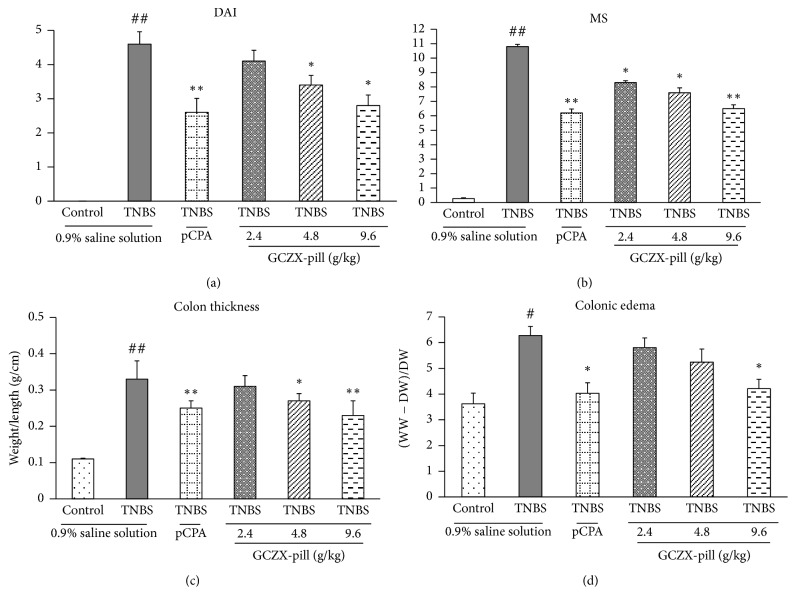
The macroscopic anti-inflammatory effects of GCZX-pill in TNBS-induced UC rats were evaluated by DAI (a), MS (b), colon thickness (c), and colonic edema (d). Data are shown as mean ± SEM (*n* = 12 per group). ^#^*P* < 0.05 and ^##^*P* < 0.01 versus the control rats; ^*∗*^*P* < 0.05 and ^*∗∗*^*P* < 0.01 versus the UC rats; one-way ANOVA followed by Bonferroni's posttest; Kruskal–Wallis analysis followed by Dunn's posttest was applied for statistical comparison of DAI and MS.

As shown in Figure 1(a), the DAIs in UC rats (TNBS group, *P* < 0.01, *n* = 12) were significantly increased when compared to that of the control rats. After pCPA treatment, the DAIs in the rats of pCPA group were significantly reduced (*P* < 0.01, *n* = 12). After GCZX-pill treatment, the DAIs in the rats of GCZX-pill groups were also significantly reduced when compared to that of the UC rats (*P* < 0.05, *n* = 12).

MS was based on the presence of adhesions, points of stenosis, mucus, erythema, and ulcers in colon, and the results were shown in Figure 1(b). Consistent with the results from DAI, the MS in UC rats (TNBS group, *P* < 0.01, *n* = 12) was significantly increased when compared to that of the control rats. After pCPA treatment, the MS in the rats of pCPA group was significantly reduced (*P* < 0.01, *n* = 12). After GCZX-pill treatment, the MS in the rats of GCZX-pill groups was also significantly reduced when compared to that of the UC rats (*P* < 0.05, *n* = 12).

The results of colon thickness were shown in Figure 1(c) and the results of colonic edema were shown in Figure 1(d). Consistent with the results from DAI and MS, both of the colon thickness and colonic edema in UC rats (*P* < 0.01 in colon thickness; *P* < 0.05 in colonic edema, *n* = 12, Dunn's test) were significantly increased when compared to that of the control rats. After pCPA or GCZX-pill treatment, the colon thickness and colonic edema in the rats of pCPA and GCZX-pill groups were significantly reduced when compared to that of the UC rats (*P* < 0.01 in colon thickness; *P* < 0.05 in colonic edema, *n* = 12).

### 3.2. Histological Evaluation of Inflammation before and after Being Treated by GCZX-Pill

**Figure 2:**
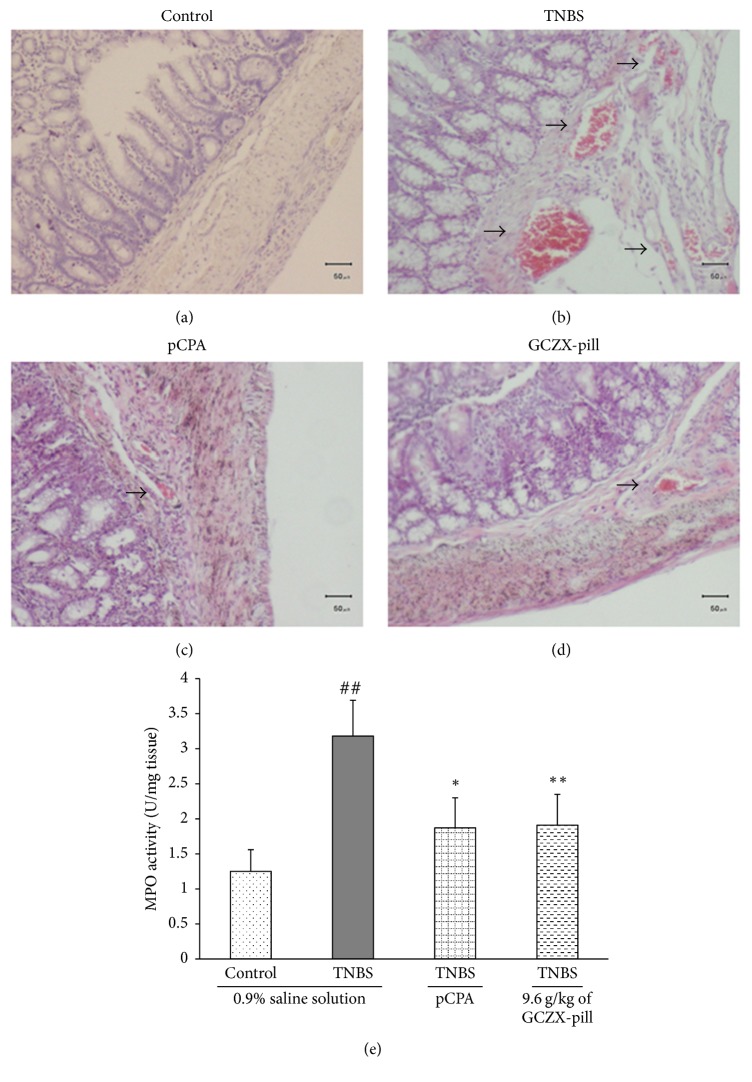
Representative hematoxylin-eosin stained sections of colonic specimens harvested from the control rats (a), from the TNBS-induced UC rats (b), from the TNBS-induced UC rats given pCPA (c), and from the TNBS-induced UC rats given GCZX-pill 9.6 g/kg (d), scale bar = 50 *μ*m; the epithelial degeneration, neutrophilic infiltration, and submucosal edema are indicated by arrows. The effect of GCZX-pill on MPO activity in TNBS-induced UC rats is shown in (e). Data are shown as mean ± SEM (*n* = 12 per group). ^##^*P* < 0.01 versus the control rats; ^*∗*^*P* < 0.05 and ^*∗∗*^*P* < 0.01 versus the UC rats; one-way ANOVA followed by Bonferroni's posttest.

The results from the hematoxylin-eosin staining of colon sections were consistent with these alterations in macroscopic evaluation of inflammation changes (Figures 2(a)–2(d)). Relative to the colon section of the control rat (Figure 2(a)), the colon section of the UC rat (Figure 2(b)) showed that severe inflammation could be detected, including epithelial degeneration, inflammatory cell infiltration, and submucosal edema. After treatment with pCPA (Figure 2(c)) or GCZX-pill (Figure 2(d)), the colon sections from the rats of pCPA group or GCZX-pill group showed a marked reduction in the tissue disruption, mucosal ulcerations, and inflammatory cell infiltration.

### 3.3. Effects of GCZX-Pill on the MPO Activity in the Colon

MPO activity is an indicator of tissue neutrophil content (Figure 2(e)). The MPO activities in UC rats were significantly increased from 1.25 ± 0.31 U/mg to 3.18 ± 0.51 U/mg (*P* < 0.01, *n* = 12) when compared to that of the control rats. After pCPA treatment, the MPO activities in the rats of pCPA group were significantly reduced to 1.87 ± 0.43 U/mg (*P* < 0.05, *n* = 12). Also, the MPO activities were significantly reduced to 1.91 ± 0.44 U/mg (*P* < 0.01, *n* = 12) by treatment with GCZX-pill when compared to that of the UC rats.

### 3.4. Effects of GCZX-Pill on the Levels of Cytokines in the Colon

**Figure 3:**
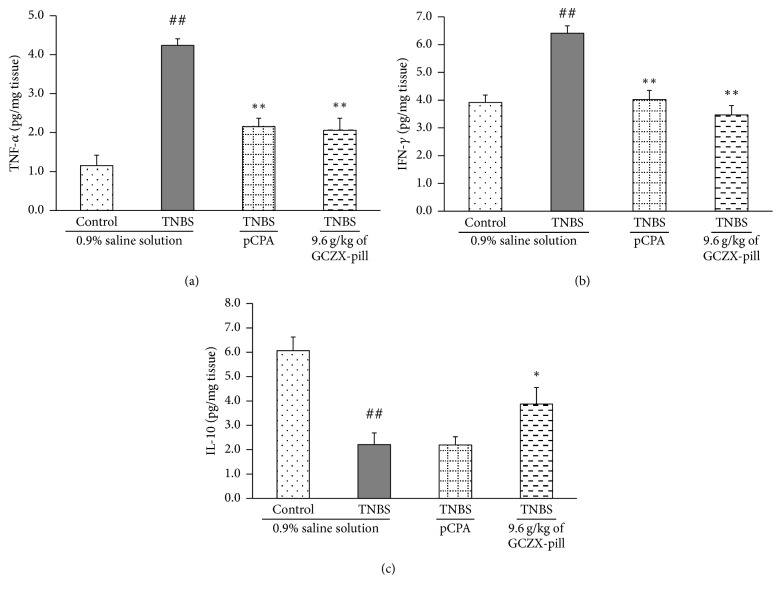
The effects of GCZX-pill on the levels of TNF*α* (a), IFN*γ* (b), and IL-10 (c) in TNBS-induced UC rats were investigated. Data are shown as mean ± SEM (*n* = 12 per group). ^##^*P* < 0.01 versus the control rats; ^*∗*^*P* < 0.05 and ^*∗∗*^*P* < 0.01 versus the UC rats; one-way ANOVA followed by Bonferroni's posttest.

The results of the changes on the levels of cytokines indicated that the levels of cytokines in the colon were associated with the development of UC (Figure 3()). The levels of TNF-*α*, IFN-*γ*, and IL-10 in the colon of UC rats (4.24 ± 0.37 pg/mg, 6.41 ± 0.27 pg/mg, and 2.21 ± 0.48 pg/mg for TNF-*α*, IFN-*γ*, and IL-10, *P* < 0.01, *n* = 12) were all significantly changed when compared to that of the control rats (1.15 ± 0.14 pg/mg, 3.92 ± 0.26 pg/mg, and 6.07 ± 0.56 pg/mg for TNF-*α*, IFN-*γ*, and IL-10). GCZX-pill treatment significantly reduced the levels of TNF-*α* and IFN-*γ* (2.06 ± 0.33 pg/mg, 3.47 ± 0.34 pg/mg for TNF-*α* and IFN-*γ*, *P* < 0.01, *n* = 12) and elevated the levels of IL-10 (3.87 ± 0.68 pg/mg, *P* < 0.05, *n* = 12) in the rats of high-dose GCZX-pill group. Similarly, pCPA treatment also significantly reduced the levels of TNF-*α* and IFN-*γ* (2.15 ± 0.41 pg/mg and 4.02 ± 0.33 pg/mg for TNF-*α* and IFN-*γ*, *P* < 0.01, *n* = 12) but did not elevate the levels of IL-10 (2.19 ± 0.34 pg/mg).

### 3.5. Effects of GCZX-Pill on Colonic EC Cell Number and TPH Expression

**Figure 4:**
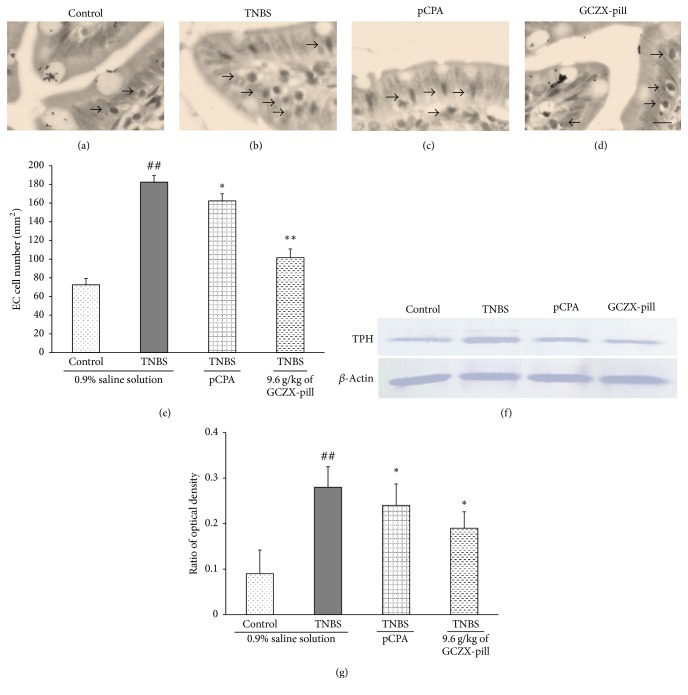
Representative Fontana-Masson stained sections of colonic EC cells specimens harvested from the control rats (a), from the TNBS-induced UC rats (b), from the TNBS-induced UC rats given pCPA (c), and from the TNBS-induced UC rats given GCZX-pill 9.6 g/kg (d), scale bar = 20 *μ*m; the EC cells are indicated by arrows. Statistical graph of EC cell density is shown in (e). Representative western blot analysis figure of colonic TPH expression (relative to beta-actin) is shown in (f). Statistical graph of quantified optical density is shown in (g). Data are shown as mean ± SEM (*n* = 12 per group). ^##^*P* < 0.01 versus the control rats; ^*∗*^*P* < 0.05 and ^*∗∗*^*P* < 0.01 versus the UC rats; one-way ANOVA followed by Bonferroni's posttest.

The effect of GCZX-pill on colonic EC cells number was investigated (Figures 4(a)–4(e)) in this study. The colonic EC cell numbers in UC rats were significantly increased from 72.5 ± 6.8 per mm^2^ to 182.4 ± 7.1 per mm^2^ (*P* < 0.01, *n* = 12) when compared to that of the control rats. After pCPA treatment, the colonic EC cells numbers in the rats of pCPA group were significantly reduced to 162.3 ± 7.6 per mm^2^ (*P* < 0.05, *n* = 12). Also, the colonic EC cells numbers were significantly reduced to 101.5 ± 9.4 per mm^2^ (*P* < 0.01, *n* = 12) by treatment with GCZX-pill when compared to that of the UC rats.

Consistent with the results from colonic EC cells number, the results (Figures 4(f) and 4(g)) also showed that colonic TPH expression in UC rats was significantly increased from 0.09 ± 0.052 to 0.28 ± 0.045 (*P* < 0.01, *n* = 12) when compared to that of the control rats. After pCPA or GCZX-pill treatment, the TPH expression in the rats of pCPA group or high-dose GCZX-pill group was significantly reduced to 0.24 ± 0.047 (pCPA group, *P* < 0.05, *n* = 12) and 0.19 ± 0.036 (high-dose GCZX-pill group, *P* < 0.05, *n* = 12) when compared to that of the UC rats.

### 3.6. Effects of GCZX-Pill on 5-HT Availability in the Colon

**Figure 5:**
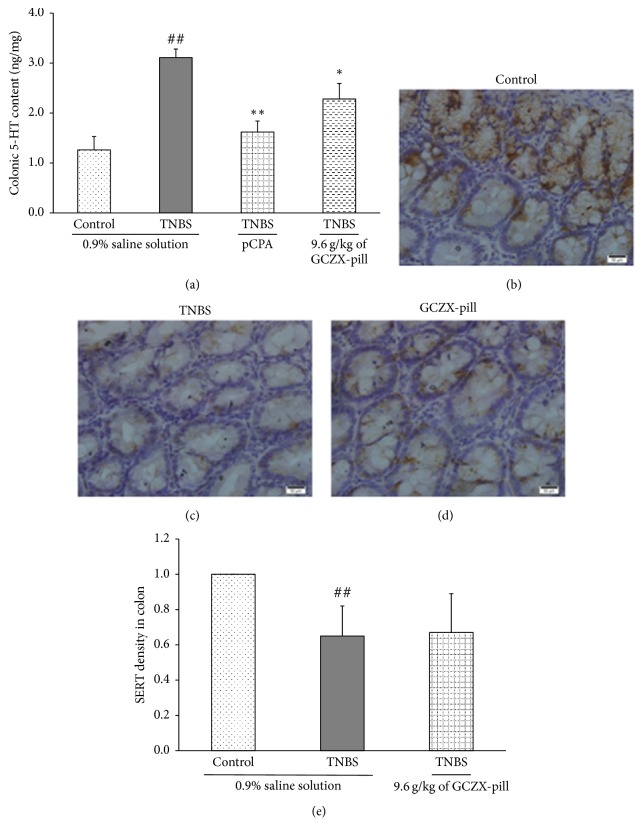
The effect of GCZX-pill on colonic 5-HT content in TNBS-induced UC rats is shown in (a). Representative immunohistochemical micrographs of colonic SERT expression specimens harvested from the control rats (b), from the TNBS-induced UC rats (c), and from the TNBS-induced UC rats given GCZX-pill 9.6 g/kg (d), scale bar = 50 *μ*m. Statistical graph of SERT expression is shown in (e). Data are shown as mean ± SEM (*n* = 12 per group). ^##^*P* < 0.01 versus the control rats; ^*∗*^*P* < 0.05 and ^*∗∗*^*P* < 0.01 versus the UC rats; one-way ANOVA followed by Bonferroni's posttest.

The effect of GCZX-pill on colonic 5-HT content was also investigated (Figure 5(a)) in this study. The colonic 5-HT content in UC rats was significantly increased from 1.26 ± 0.27 ng/mg to 3.11 ± 0.17 ng/mg (*P* < 0.01, *n* = 12) when compared to that of the control rats. After pCPA treatment, the colonic 5-HT content in the rats of pCPA group was significantly reduced to 1.62 ± 0.22 ng/mg (*P* < 0.01, *n* = 12). Also, the colonic 5-HT content was significantly reduced to 2.28 ± 0.31 ng/mg (*P* < 0.05, *n* = 12) by treatment with GCZX-pill when compared to that of the UC rats.

The colonic intensity of SERT immunoreactivity (Figures 5(b)–5(e)) in UC rats was significantly decreased when compared to that of the control rats (*P* < 0.01, *n* = 12). After treatment with GCZX-pill, there were no significant differences in the expression of SERT immunoreactive intensity between UC rats and GCZX-pill-treated rats, suggesting that GCZX-pill treatment has little effect on the SERT expression in UC rats.

## 4. Discussion

The results from this study indicated an important effect for GCZX-pill in inhibiting TNBS-induced UC. And the anti-inflammatory effect of GCZX-pill administration indicated that GCZX-pill significantly attenuated colitis. Meanwhile, the changes of EC cell numbers, colonic TPH expression, and 5-HT availability induced by GCZX-pill at colon were also indicated here.

To evaluate the anti-inflammatory effect of GCZX-pill, the well-established model of TNBS-induced colitis in rats which has resemblance to UC was used. In this study, GCZX-pill efficiently alleviated TNBS-induced UC. TNBS is widely used for studying ulcerative colitis because the TNBS-induced UC model is symptomatically, morphologically, and histopathologically very similar to that of human IBD [24]. When TNBS couples with high molecular weight proteins it can elicit significant immunologic responses by rendering those proteins immunogenic to the host immune system and induces diffuse colonic inflammation, which is characterized by increased leukocyte infiltration, edema, and ulceration [35, 36]. Experimentally, TNBS is dissolved in alcohol and is delivered intrarectally in rodents to induce colitis. Alcohol not only serves as a solvent or carrier, but also aids in inducing gut inflammation by breaking the mucosal barrier [37, 38]. However, TNBS also has inherent limitations that it does not recapitulate the exact mechanisms that underlie its pathogenesis and specifically the role of gut microbiota [39]. The symptoms such as the attenuation of weight loss, abnormal defecation, rectal bleeding, adhesion, intraluminal hemorrhage, colonic thickness, and colonic edema were alleviated by GCZX-pill. Also, GCZX-pill reduced MPO activity, a marker of tissue neutrophil content.

As expected, EC cell hyperplasia and high-concentration of 5-HT were induced by TNBS which is consistent with the characteristic of UC. 5-HT was synthesized and released from enterochromaffin (EC) cells [13]. As a neurotransmitter, 5-HT plays an important role in the gastrointestinal tract. There are many serotonergic receptors that have been found on various immune cells such as B and T lymphocytes, monocytes, macrophage, and dendritic cells [40]. In addition, 5-HT is also a chemotactic molecule for eosinophils, dendritic cells, and mast cells. Therefore, it is suggested that 5-HT plays an important role in influencing the immune system [41]. Results from this study showed that GCZX-pill treatment significantly alleviated inflammation in UC rats, and this effect was concomitant with the decreased colonic EC cell number and 5-HT concentration. TPH in EC cells is the rate-limiting enzyme in the 5-HT synthesis process, and the pCPA is one of the inhibitors of TPH [42, 43]. Results from this study show that pCPA depleted seriously the colonic 5-HT concentration and a well anti-inflammatory effect in TNBS-induced UC is shown. Therefore, all these results indicate that the decreased EC cell number and 5-HT concentration induced by GCZX-pill treatment may be responsible for attenuated the inflammation in UC rats.

The results from this study indicated that the levels of TNF-*α*, IFN-*γ*, and IL-10 in the colon were changed by treatment with GCZX-pill. Past study indicated that the levels of Th1 or Th2 inflammatory cytokines such as TNF-*α*, IFN-*γ*, and IL-10 play important roles in Th1/Th2 balance and expansion of CD4^+^ T cells, and the classical Th1/Th2 pathways are thought to play a critical role in UC pathogenesis. In addition, the past reports indicated that EC cell hyperplasia is considered to have close correlation with CD4^+^ T lymphocytes, especially the Th1/Th2 balance [14, 15, 44]. Therefore, the inflammation and hyperplastic colonic EC cells that were attenuated by GCZX-pill may be mediated by changing the levels of TNF-*α*, IFN-*γ*, and IL-10.

A similar phenomenon was observed where the hyperplastic colonic EC cells were reduced by pCPA. As an inhibitor of TPH, pCPA significantly reduced the colonic 5-HT content. Past studies indicated the reduced severity of colitis in TPH1^−/−^ mice as compared with wild-type mice after dextran sodium sulfate- and dinitrobenzenesulfonic acid-colitis. Restoration of 5-HT in TPH1^−/−^ mice by administration of a 5-HT precursor (5-hydroxy-l-tryptophan) enhanced the severity of colitis. Therefore, the inflammation could be attenuated via decreasing the colonic 5-HT content [40]. In addition, besides its well-characterized function as a neurotransmitter, 5-HT has been reported to be a potent immunoregulator. 5-HT has been reported to be a potent regulator of cytokine secretion in different kinds of cells [45]. Past studies have shown an important immunoendocrine axis in the gut in an enteric infection-induced model of gut inflammation, where secretory products from CD4^+^ T cells interact with EC cells or their precursors to enhance 5-HT production in the gut [27, 46]. Therefore, it is not hard to understand that pCPA, as an inhibitor of TPH reducing the hyperplastic colonic EC cells, may be mediated by reducing colonic 5-HT content.

The serotonin reuptake transporter (SERT) plays important roles in 5-HT recycle in the colon tissue [12, 47]. The effect of GCZX-pill on the expression of SERT was also investigated in this study. Results from this study were consistent with that of the past studies which showed that SERT expression is downregulated in TNBS-induced colitis. Unfortunately, after treatment with GCZX-pill, there were no significant differences in the expression of SERT immunoreactive intensity between UC rats and GCZX-pill-treated rats. Therefore, this result indicated that GCZX-pill did not act on the recycling of 5-HT.

It is well known that most traditional Chinese medicine remedies are formulated by using individual herbs in combination. Under the guidance of traditional theory, the different herbs of certain formula are thought to increase therapeutic efficacy and reduce adverse effects simultaneously through multiple targets and biological pathways [48]. Results from this study indicated that the anti-inflammatory effect of GCZX-pill may be mediated by reducing colonic EC cell number, 5-HT content, and TPH expression, but not SERT expression, in UC rats. In conclusion, this study demonstrated that the GCZX-pill can attenuate the inflammation in UC rats. The anti-inflammatory effect of the GCZX-pill may be mediated by reducing colonic EC cell hyperplasia and 5-HT availability.

## References

[1] Matricon J., Barnich N., Ardid D. (2010). Immunopathogenesis of inflammatory bowel disease. *Self/Nonself—Immune Recognition and Signaling*.

[2] Zhang Y.-Z., Li Y.-Y. (2014). Inflammatory bowel disease: pathogenesis. *World Journal of Gastroenterology*.

[3] Kopylov U., Ben-Horin S., Seidman E. (2014). Therapeutic drug monitoring in inflammatory bowel disease. *Annals of Gastroenterology*.

[4] Ardizzone S., Cassinotti A., Manes G., Porro G. B. (2010). Immunomodulators for all patients with inflammatory bowel disease?. *Therapeutic Advances in Gastroenterology*.

[5] Bongartz T., Sutton A. J., Sweeting M. J., Buchan I., Matteson E. L., Montori V. (2006). Anti-TNF antibody therapy in rheumatoid arthritis and the risk of serious infections and malignancies: systematic review and meta-analysis of rare harmful effects in randomized controlled trials. *Journal of the American Medical Association*.

[6] Targownik L. E., Bernstein C. N. (2013). Infectious and malignant complications of tnf inhibitor therapy in ibd. *American Journal of Gastroenterology*.

[7] Sałaga M., Zatorski H., Sobczak M., Chen C., Fichna J. (2014). Chinese herbal medicines in the treatment of IBD and colorectal cancer: a review. *Current Treatment Options in Oncology*.

[8] Wan P., Chen H., Guo Y., Bai A.-P. (2014). Advances in treatment of ulcerative colitis with herbs: From bench to bedside. *World Journal of Gastroenterology*.

[9] Coates M. D., Mahoney C. R., Linden D. R. (2004). Molecular defects in mucosal serotonin content and decreased serotonin reuptake transporter in ulcerative colitis and irritable bowel syndrome. *Gastroenterology*.

[10] Magro F., Vieira-Coelho M. A., Fraga S. (2002). Impaired synthesis or cellular storage of norepinephrine, dopamine, and 5-hydroxytryptamine in human inflammatory bowel disease. *Digestive Diseases and Sciences*.

[11] De Ponti F. (2004). Pharmacology of serotonin: what a clinician should know. *Gut*.

[12] Linden D. R., Chen J.-X., Gershon M. D., Sharkey K. A., Mawe G. M. (2003). Serotonin availability is increased in mucosa of guinea pigs with TNBS-induced colitis. *American Journal of Physiology—Gastrointestinal and Liver Physiology*.

[13] El-Salhy M., Danielsson Å., Stenling R., Grimelius L. (1997). Colonic endocrine cells in inflammatory bowel disease. *Journal of Internal Medicine*.

[14] Wang H., Steeds J., Motomura Y. (2007). CD4+ T cell-mediated immunological control of enterochromaffin cell hyperplasia and 5-hydroxytryptamine production in enteric infection. *Gut*.

[15] Motomura Y., Ghia J.-E., Wang H. (2008). Enterochromaffin cell and 5-hydroxytryptamine responses to the same infectious agent differ in Th1 and Th2 dominant environments. *Gut*.

[16] Wang X., Liu L., Sun J. (2014). Application of superfine grinding technology in the production of Guchang Zhixie pills. *Modern Traditional Chinese Medicine*.

[17] Liu Y., Song Z., Zhang Y. (2015). SQFM methods based on HPLC- DAD with five wavelengths for fingerprint determination of Guchang Zhixie pill and powders. *Chinese Journal of Experimental Traditional Medical Formulae*.

[18] Liu Y., Song Z., Zhang Y. (2015). Application of Plackett-Burman-Central composite design for fingerprint analysis of Gu-Chang-Zhi-Xie pill by HPLC-DAD approach. *Modern Chinese Medicine*.

[19] Yuan W. (2013). Clinical research on treating diarrhea-predominant irritable bowel syndrome with the Guchang Zhixie pills. *Clinical Journal of Chinese medicine*.

[20] Liu Y. (2016). Clinical observation of Guchang Zhixie pills combined with compound glutamin entersoluble capsules in treatment of ulcerative colitis. *Drugs & Clinic*.

[21] Huang H., Ji S. (2006). The therapeutic effect of combining Guchangzhixie Wan with Smecta in treating irritable bowel syndrome of diarrheal type. *Practical Clinical Journal of Integrated Traditional Chinese and Western Medicine*.

[22] Wu H., Chen Z. (2014). Efficacy of Guchang Zhixie pill and sulfasalazine in treatment of paients with ulcerative colitis and influence of IL-2 and IL-8 in serum. *Liaoning Journal of Traditional Chinese Medicine*.

[23] Qin H.-Y., Wu J. C. Y., Tong X.-D., Sung J. J. Y., Xu H.-X., Bian Z.-X. (2011). Systematic review of animal models of post-infectious/post-inflammatory irritable bowel syndrome. *Journal of Gastroenterology*.

[24] De Almeida A. B. A., Sánchez-Hidalgo M., Martín A. R. (2013). Anti-inflammatory intestinal activity of Arctium lappa L. (Asteraceae) in TNBS colitis model. *Journal of Ethnopharmacology*.

[25] Cooper H. S., Murthy S. N., Shah R. S., Sedergran D. J. (1993). Clinicopathologic study of dextran sulfate sodium experimental murine colitis. *Laboratory Investigation*.

[26] Wallace J. L., MacNaughton W. K., Morris G. P., Beck P. L. (1989). Inhibition of leukotriene synthesis markedly accelerates healing in a rat model of inflammatory bowel disease. *Gastroenterology*.

[27] Bischoff S. C., Mailer R., Pabst O. (2009). Role of serotonin in intestinal inflammation: Knockout of serotonin reuptake transporter exacerbates 2,4,6-trinitrobenzene sulfonic acid colitis in mice. *American Journal of Physiology—Gastrointestinal and Liver Physiology*.

[28] Moore-Olufemi S. D., Kozar R. A., Moore F. A. (2005). Ischemic preconditioning protects against gut dysfunction and mucosal injury after ischemia/reperfusion injury. *Shock*.

[29] Khan W. I., Blennerhasset P. A., Varghese A. K. (2002). Intestinal nematode infection ameliorates experimental colitis in mice. *Infection and Immunity*.

[30] Krawisz J. E., Sharon P., Stenson W. F. (1984). Quantitative assay for acute intestinal inflammation based on myeloperoxidase activity. Assessment of inflammation in rat and hamster models. *Gastroenterology*.

[31] Grisham M. B., Benoit J. N., Granger D. N. (1990). Assessment of leukocyte involvement during ischemia and reperfusion of intestine. *Methods in Enzymology*.

[32] Zhu X., Liu Z., Qu H. (2016). The effect and mechanism of electroacupuncture at LI11 and ST37 on constipation in a rat model. *Acupuncture in Medicine*.

[33] Zhu X., Liu Z., Niu W. (2016). Effects of electroacupuncture at ST25 and BL25 in a *Sennae*-induced rat model of diarrhoea-predominant irritable bowel syndrome. *Acupuncture in Medicine*.

[34] Zhu X., Shinohara H., Miyatake R., Hohsaka T. (2016). Novel biosensor system model based on fluorescence quenching by a fluorescent streptavidin and carbazole-labeled biotin. *Journal of Molecular Recognition*.

[35] Elson C. O., Sartor R. B., Tennyson G. S., Riddell R. H. (1995). Experimental models of inflammatory bowel disease. *Gastroenterology*.

[36] Isik F., Tunali Akbay T., Yarat A. (2011). Protective effects of black cumin (*Nigella sativa*) oil on TNBS-induced experimental colitis in rats. *Digestive Diseases and Sciences*.

[37] Neurath M. F., Fuss I., Kelsall B. L., Stüber E., Strober W. (1995). Antibodies to interleukin 12 abrogate established experimental colitis in mice. *Journal of Experimental Medicine*.

[38] Ikeda M., Takeshima F., Isomoto H. (2008). Simvastatin attenuates trinitrobenzene sulfonic acid-induced colitis, but not oxazalone-induced colitis. *Digestive Diseases and Sciences*.

[39] Antoniou E., Margonis G. A., Angelou A. (2016). The TNBS-induced colitis animal model: an overview. *Annals of Medicine and Surgery*.

[40] Ghia J.-E., Li N., Wang H. (2009). Serotonin has a key role in pathogenesis of experimental colitis. *Gastroenterology*.

[41] Cloëz-Tayarani I., Changeux J.-P. (2007). Nicotine and serotonin in immune regulation and inflammatory processes: a perspective. *Journal of Leukocyte Biology*.

[42] Walther D. J., Peter J.-U., Bashammakh S. (2003). Synthesis of serotonin by a second tryptophan hydroxylase isoform. *Science*.

[43] Walther D. J., Bader M. (2003). A unique central tryptophan hydroxylase isoform. *Biochemical Pharmacology*.

[44] Strober W., Fuss I. J. (2011). Proinflammatory cytokines in the pathogenesis of inflammatory bowel diseases. *Gastroenterology*.

[45] Müller T., Dürk T., Blumenthal B. (2009). 5-hydroxytryptamine modulates migration, cytokine and chemokine release and T-cell priming capacity of dendritic cells in vitro and in vivo. *PLoS ONE*.

[46] Haub S., Ritze Y., Bergheim I., Pabst O., Gershon M. D., Bischoff S. C. (2010). Enhancement of intestinal inflammation in mice lacking interleukin 10 by deletion of the serotonin reuptake transporter. *Neurogastroenterology and Motility*.

[47] Hansen M. B. (2003). Neurohumoral control of gastrointestinal motility. *Physiological Research*.

[48] Wang L., Zhou G.-B., Liu P. (2008). Dissection of mechanisms of Chinese medicinal formula Realgar-Indigo naturalis as an effective treatment for promyelocytic leukemia. *Proceedings of the National Academy of Sciences of the United States of America*.

